# Sensor Fusion for Enhancing Motion Capture: Integrating Optical and Inertial Motion Capture Systems †

**DOI:** 10.3390/s25154680

**Published:** 2025-07-29

**Authors:** Hailey N. Hicks, Howard Chen, Sara A. Harper

**Affiliations:** 1 Industrial & Systems Engineering and Engineering Management Department, University of Alabama in Huntsville, Huntsville, AL 35899, USA; hc0060@uah.edu; 2 Kinesiology Department, University of Alabama in Huntsville, Huntsville, AL 35899, USA; sah0075@uah.edu

**Keywords:** sensor fusion, biomechanical motion analysis, field-based testing, optical motion capture, inertial motion capture

## Abstract

This study aimed to create and evaluate an optimization-based sensor fusion algorithm that combines Optical Motion Capture (OMC) and Inertial Motion Capture (IMC) measurements to provide a more efficient and reliable gap-filling process for OMC measurements to be used for future research. The proposed algorithm takes the first and last frame of OMC data and fills the rest with gyroscope data from the IMC. The algorithm was validated using data from twelve participants who performed a hand cycling task with an inertial measurement unit (IMU) placed on their hand, forearm, and upper arm. The OMC tracked a cluster of reflective markers that were placed on top of each IMU. The proposed algorithm was evaluated with simulated gaps of up to five minutes. Average total root-mean-square errors (RMSE) of <1.8° across a 5 min duration were observed for all sensor placements for the cyclic upper limb motion pattern used in this study. The results demonstrated that the fusion of these two sensing modalities is feasible and shines light on the possibility of more field-based studies for human motion analysis.

## 1. Introduction and Background

Optical Motion Capture Systems (OMCs) are used for human motion analysis for their accuracy and are considered the gold standard [1]. OMCs utilize high-resolution cameras to track the position of reflective markers [2]. Orientation can be calculated if a minimum of three markers is attached to a rigid body (e.g., body segment). While OMCs are considered the gold standard for human motion analysis, factors such as number of cameras, movement conditions, analysis height, and capture volume can impact the accuracy of the results [3]. Nevertheless, the data values are still within a millimeter (mm) of true values [4]. While OMCs are useful for collecting highly accurate data, they also have a variety of drawbacks that can limit their utility. Multiple studies have shown that capture volume and test setting can have direct effects on the accuracy of the data collected [5,6].

Arguably, the biggest limitation of OMCs is marker occlusion. Each marker needs to be captured by at least two cameras to determine its position in three-dimensional space. If this does not occur, or if the marker(s) get blocked, gaps will be present in the dataset. While gaps in missing data can be filled in after collection, this time-intensive post-processing technique can lead to values that stray from the truth [7]. Although more cameras will help alleviate marker occlusion, it comes with increased system cost and system setup time. Each OMC has a method to address and fill the gaps in the data, but most require human involvement, which is a time-intensive and imprecise process. Furthermore, the predicted values can stray from the true results [7]. A study conducted by Gomes et al. (2021) further demonstrated the need for more advanced gap-filling strategies by directly comparing their automated solution with three OMC strategies—spline, pattern, and rigid body fill—and their solution outperformed all of the OMC’s methods [8]. In light of this issue, many researchers have proposed methods to more accurately fill in the gaps when they occur, aside from the sole use of OMCs [9,10,11].

Human motion can be alternatively captured using Inertial Motion Capture Systems (IMCs), which use wearable sensors that are attached to various body segments of a given individual [12]. Each of these wearable sensors is called an inertial measurement unit (IMU) and contains a gyroscope, accelerometer, and a magnetometer. The measurements from these sensors allow an IMU to establish orientation of any given body segment by integrating angular velocity from the gyroscope with respect to time and correcting any resulting errors using accelerometer and magnetometer measurements [13,14]. Since IMUs do not involve reflective markers, there are no concerns associated with marker occlusion. Studies have shown that even low-cost IMUs are reliable for ground navigation, human activity recognition, and human motion analysis [15,16].

Although IMUs can be advantageous for analyzing movement, they have their own limitations. The accuracy of IMUs is adversely affected by movement, presence of magnetic disturbances, and gyroscopic drift [17]. Specifically, since the gyroscope measurements are integrated with respect to time, errors in sensor measurements are also integrated and will also compound over time, resulting in drift [17]. Other limitations to IMUs are bias stability and noise characteristics. These factors are inevitable when dealing with IMUs and must be taken into consideration [18]. One way to mitigate this is to measure the bias using a static trial. Other methods involve using the beginning of a dynamic trial as a reference for orientation and estimation error to be referenced throughout [19]. To our knowledge, few studies have examined the use of IMUs to gap-fill OMC data. For this application, the gyroscopic drift is mitigated by combining gyroscope measurements from the IMU with OMC-derived orientation rather than accelerometer and magnetometer measurements from the IMU, thus alleviating the increased errors due to effects of movement and presence of magnetic disturbance on IMU measurement accuracy.

Finding a way to combine both IMCs and OMCs to overcome their respective limitations for the most accurate data collection is an ever-growing area of research. One method to accomplish this comes through sensor fusion. Sensor fusion involves taking data from different sensing modalities and combining them to eliminate some of the errors that arise from solely relying on one source or the other [20]. Most sensor fusion algorithms focus solely on improving IMCs. There are currently only a few studies that implement both IMC and OMC measurements by creating custom algorithms using various sensor fusion methods. Yang et al. (2021) fused gyroscope and OMC data for more precise orientation, yet human participants were only included in one short trial (<1 min), and only small marker occlusion gaps were filled [21]. Enayati et al. (2015) used a Kalman filter algorithm; however, human participants were not included in the data collection, and the authors emphasized only filling small data gaps [22]. Consequently, the ability of this approach to mitigate marker occlusion with human participants over longer time periods (i.e., minutes) is unknown [22]. Our previous study [23] demonstrated the capability of the proposed algorithm for combining IMC and OMC measurements for the hand. This study provided an in-depth description of the proposed algorithm and extended the analysis to include the upper arm and forearm locations.

Specifically, this study aimed to create and evaluate an optimization-based sensor fusion algorithm that combines OMC and IMC measurements to provide a more efficient and reliable gap-filling process for OMC measurements to be used for future research. The proposed algorithm takes the first and last frame of OMC data and fills the rest with IMU gyroscope data. We hypothesized that each sensor placement would have increased total error as time increases (min 1, 2, and 5), with the sensor placement having no effect on the error outputs.

## 2. Materials and Methods

### 2.1. Theoretical Background

#### 2.1.1. Spatial Orientation

A rotation matrix ($R^{nb}$) is a 3 × 3 matrix that describes spatial orientation and can be defined as follows:

(1) $$x^{n}=R^{nb}x^{b\;}$$

where ($x^{b}$) represents a vector in the local (body) frame, and ($x^{n}$) represents the corresponding vector in the navigation (global) frame. Spatial orientation can alternatively be described using a 4 × 1 quaternion vector q consisting of scalar component $q_{0}$ and vector component $q_{v}$:

(2) $$q=\left[\begin{array}{c} q_{0}\\ q_{1}\\ q_{2}\\ q_{3} \end{array}\right]=\left[\begin{array}{c} q_{0}\\ q_{v} \end{array}\right].$$

The quaternion vector $q^{nb}$ can be used to transform from $x^{b}$ to $x^{n}$ as follows:

(3) $$\left[\begin{array}{c} 0\\ x^{n} \end{array}\right]=q^{nb}☉\left[\begin{array}{c} 0\\ x^{b} \end{array}\right]☉{\left(q^{nb}\right)}^{C}$$

where ☉ is the quaternion product, and ${(q^{nb})\;}^{C}$ is the quaternion conjugate of $q^{nb}$. The quaternion product can be calculated as follows:

(4) $$p\;☉q=\left(\begin{array}{ccc} p_{0}q_{0} & - & p_{v}q_{v}\\ p_{0}q_{v} & + & p_{v}\times q_{v} \end{array}\right)=\;p^{L}q=\;q^{R}p$$

where

(5) $$p^{L}≜\left(\begin{array}{cc} p_{0} & -p_{v}^{T}\\ p_{v} & p_{0}I_{3}+\left[q_{v}\times \right] \end{array}\right),\;\;\;\;\;\;\;q^{R}≜\left(\begin{array}{cc} q_{0} & -q_{v}^{T}\\ q_{v} & q_{0}I_{3}-\left[q_{v}\times \right] \end{array}\right)$$

and $I_{3}$ is a 3 × 3 identity matrix, [×] is the skew symmetric operator, and T represents the transpose of the matrix. The quaternion conjugate is defined as follows:

(6) $$q^{C}=(\begin{array}{cc} q_{0} & -q_{v}^{T}{)}^{T} \end{array}$$

It can be used to transform $x^{n}$ to $x^{b}$ as follows:

(7) $$q^{bn}={\left(q^{nb}\right)}^{C}\;.$$

#### 2.1.2. Gyroscope Measurement Models

For the IMC, the IMU gyroscope measurements are of interest for this study since the orientation from the OMC is used to ‘correct’ for gyroscopic drift instead of orientation derived from the accelerometer and magnetometer. The gyroscope measures angular velocity at time t. The gyroscope measurements consider the model below:

(8) $$y_{\omega ,t}=\omega_{nb,t}^{b}+b_{\omega ,t}^{b}+e_{\omega ,t}^{b}$$

where the measured angular velocity is represented by $y_{\omega ,t}$, nominal angular velocity is represented by $\omega_{nb,t}^{b}$, bias is represented by$\;b_{\omega ,t}^{b}$, and white noise is represented by $e_{\omega ,t}^{b}$. Both $b_{\omega ,t}^{b}$ and $e_{\omega ,t}^{b}$ are assumed to follow a zero-mean gaussian distribution. Other important assumptions to note include (i) the fact that there are no other unknown parameters in the model and (ii) that the noise of each axis is independent.

Orientation can be calculated using gyroscope measurements as follows, where ΔT is the sampling period:

(9) $$q_{t+1}^{nb}=q_{t}^{nb}☉{exp}_{q}(\frac{\Delta T}{2}\left(y_{\omega ,t}-b_{\omega }-e_{\omega ,t}\right))$$

where

(10) $${exp}_{q}\left(\eta \right)\approx \left[\begin{array}{c} 1\\ \eta \end{array}\right].$$

### 2.2. Solving for the Optimization Function

Given a residual function ε, the parameters of interest at iteration k (${\hat{x}}^{k}$) can be obtained by iterating the following function until convergence:

(11) $${\hat{x}}_{1:N}^{\left(k+1\right)}={\hat{x}}_{1:N}^{\left(k\right)}-\beta^{\left(k\right)}{\left(\hat{H}\left({\hat{x}}_{1:N}^{\left(k\right)}\right)\right)}^{-1}G\left({\hat{x}}_{1:N}^{\left(k\right)}\right)$$

where $\beta^{k}$ is the step length, $\hat{H}$ is the Hessian approximation, and G is the gradient, which are defined as:

(12) $$\hat{H}\approx J^{T}J$$

(13) $$G=J^{T}\varepsilon$$

and

(14) $$J_{\left(x1:N\right)}=\left(\begin{array}{ccc} \frac{\partial \varepsilon_{1}}{\partial x_{1}} & \ldots & \frac{\partial \varepsilon_{1}}{\partial x_{N}}\\ \vdots & & \vdots \\ \frac{\partial \varepsilon_{Mn_{\varepsilon }}}{\partial x_{1}} & \ldots & \frac{\partial \varepsilon_{Mn_{\varepsilon }}}{\partial x_{N}} \end{array}\right).$$

#### 2.2.1. IMC-OMC Fusion

An optimization-based sensor fusion method from Kok et al. (2017) was adopted to combine the gyroscope data from the IMU with the OMC-derived orientation [24]. Specifically, given a sequence of angular velocity measurements from the gyroscope contained within the IMU, as well as the OMC-derived orientation from the OMC for the first and last frames of the sequence, the algorithm will estimate the spatial orientation and the gyroscope bias under the assumption that it does not change during a given time period.

Given a sequence of measurements from time t = 1 to t = N, the optimization problem was formulated as

(15) $${\hat{x}}_{1:N}=\underset{x_{1:N}}{\min}\left({\left\|e_{q,1}\right\|}^{2}+{\left\|e_{q,N}\right\|}^{2}+\sum_{T=1}^{n}{\left\|e_{\omega ,t}\right\|}^{2}\right)$$

where ${\hat{x}}_{1:N}$ contains the parameters to be estimated, consisting of $q_{t}^{nb}$ and $b_{\omega ,t}$. The residual functions for the incorporation of OMC-derived orientation and gyroscope measurements are given by $e_{q,t}$ and $e_{\omega ,t}$, respectively, and are defined as follows:

(16) $$e_{q,t}=2\log_{q}\left(q_{t}^{nb}☉{\left({\overset{ˇ}{q}}_{t}^{nb}\right)}^{C}\right)$$

(17) $$e_{\omega ,t}=\frac{2}{\Delta T}\log_{q}\left({\left(q_{t}^{nb}\right)}^{C}☉\left(q_{t+1}^{nb}\right)\right)-{(y}_{\omega ,t}-b_{\omega ,t})$$

where ${\overset{ˇ}{q}}_{t}^{nb}$ is the OMC-derived orientation described using quaternions, and

(18) $$\log_{q}\left(q\right)\approx q_{v}.$$

Instead of solving for $q_{t}^{nb}$ and $b_{\omega }$ directly, orientation deviation ($\eta_{t})$ and gyroscope bias deviation $(\delta b_{\omega }$) were solved and subsequently used to ‘correct’ $q_{t}^{nb}$ and $b_{\omega }$, respectively, for numerical stability [24]. The residual function was re-parameterized as follows:

(19) $$e_{q,t}=2\log_{q}\left(\exp_{q}\left(\frac{\eta_{t}}{2}\right)☉q_{t}^{nb}☉{\left({\overset{ˇ}{q}}_{t}^{nb}\right)}^{C}\right)$$

(20) $$e_{\omega ,t}=\frac{2}{\Delta T}\log_{q}\left({\left(\exp_{q}\left(\frac{\eta_{t}}{2}\right)☉q_{t}^{nb}\right)}^{C}☉\left(\exp_{q}\left(\frac{\eta_{t+1}}{2}\right)☉q_{t+1}^{nb}\right)\right)-{(y}_{\omega ,t}-b_{\omega ,t}-\delta_{b\omega ,t})$$

At every iteration, $q_{t}^{nb}$ and $b_{\omega ,t}$ is updated:

(21) $$q_{t}^{nb}=\exp_{q}\left(\frac{\eta_{t}}{2}\right)☉q_{t}^{nb}$$

(22) $$b_{\omega }=b_{\omega }+\delta_{b\omega ,t}$$

$\eta_{t}$ and $\delta_{b\omega ,t}$ are set to zeros. Since $\eta_{t}$ and $\delta_{b\omega ,t}$ are set to zeros, Equations (16) and (17) can be used instead of Equations (19) and (20). Equation (15) become

(23) $${\hat{x}}_{1:N}^{\left(k+1\right)}=-\beta^{\left(k\right)}{\left(\hat{H}\left({\hat{x}}_{1:N}^{\left(k\right)}\right)\right)}^{-1}G\left({\hat{x}}_{1:N}^{\left(k\right)}\right).$$

The overall residual function ε is defined as

(24) $$\varepsilon_{1:N}=\left[\begin{array}{c} e_{\omega ,1:N}\\ e_{q,1}\\ e_{q,N} \end{array}\right]$$

and the corresponding Jacobian of the OMC residual with respect to $\eta_{t}$ is calculated using the following:

(25) $$\frac{\partial e_{\eta ,i}}{\partial \eta_{i}^{n}}=\frac{\partial {log}_{q}\left(q\right)}{\partial q}\;\cdot \;{(\tilde{q}}_{i}^{nb}\;ʘ\;{\overset{ˇ}{q}}_{i}^{nb}{)}^{R}\;\cdot \;\frac{\partial {exp}_{q}\left(\eta_{i}^{n}\right)}{\partial \left(\eta_{i}^{n}\right)}$$

Similarly, the Jacobian of the gyroscope residual with respect to $\eta_{t},$ $\eta_{t+1}$, and $\delta_{b\omega }$ is calculated as

(26) $$\frac{\partial e_{\omega ,t}}{\partial \eta_{t}^{n}}=\frac{1}{\Delta T}\cdot \frac{\partial {log}_{q}\left(q\right)}{\partial q}\cdot {\left({\tilde{q}}_{t}^{bn}\right)}^{L}\cdot {\left({\tilde{q}}_{t+1}^{nb}\right)}^{R}\cdot \;\frac{\partial {(exp}_{q}({\eta_{t}^{n}))}^{C}}{\partial {exp}_{q}\left(\eta_{t}^{n}\right)}\cdot \frac{\partial {exp}_{q}\left(\eta_{t}^{n}\right)}{\partial \left(\eta_{t}^{n}\right)}$$

(27) $$\frac{\partial e_{\omega ,t}}{\partial \eta_{t+1}^{n}}=\frac{1}{\Delta T}\cdot \frac{\partial {log}_{q}\left(q\right)}{\partial q}\cdot {\left({\tilde{q}}_{t}^{bn}\right)}^{L}\cdot {\left({\tilde{q}}_{t+1}^{nb}\right)}^{R}\cdot \frac{\partial {exp}_{q}\left(\eta_{1+1}^{n}\right)}{\partial \eta_{t+1}^{n}}$$

(28) $$\frac{\partial e_{\omega ,t}}{\partial \delta_{b\omega }}=I_{3}$$

where

(29) $$\frac{\partial {log}_{q}\left(q\right)}{\partial q}\;\approx \;{\left(\begin{array}{c} 0_{1\times 3}\\ I_{3} \end{array}\right)}^{T},\;\frac{\partial ({{exp}_{q}(\eta ))}^{C}}{\partial {exp}_{q}\left(\eta \right)}=\left(\begin{array}{cc} 1 & 0_{1\times 3}\\ 0_{3\times 1} & -I_{3} \end{array}\right),\;\;\;\;\frac{\partial {exp}_{q}\left(\eta \right)}{\partial \eta }\;\approx \;\left(\begin{array}{c} 0_{1\times 3}\\ I_{3} \end{array}\right).$$

The resulting algorithm is summarized in Algorithm 1 [25].
**Algorithm 1** Calculating orientation using IMU and OMC measurements**Inputs:** Gyroscope data from t = 1 to t = N, OMC orientation at t = 1 and t = N
**Output:** Estimate of orientation from t = 1 to t = N1. **Initialize** $q^{nb}$ to [1 0 0 0] for T = 1 to T = N
2. **While** convergence criteria is not met **do:**
  (A) Calculate the residual (Equation (24))
  (B) Calculate the corresponding Jacobians (Equations (26)–(28))
  (C) calculate $\eta_{t}$$\;\mathrm{and}\;\delta b_{\omega }$ through optimization (Equation (23))
  (D) Correct the orientation (Equation (21))
  (E) Correct the gyroscope bias (Equation (22))

#### 2.2.2. OMC-IMU Alignment

The IMU data were temporally aligned to the OMC using cross-correlation. Specifically, the OMC-derived orientation was differenced to obtain angular velocities in the body frame. The Euclidean norm of each sample of the OMC and gyroscope-derived angular velocity was subsequently calculated. The temporal sensor alignment was accomplished by first calculating the cross-correlation of the normed velocities and then determining the lag value corresponding to the largest spike in the cross-correlation. Note that the normalized angular velocities were used for this purpose to determine temporal alignment independent of rotational alignment between the OMC and IMU. The local frame of the IMU was subsequently aligned to the global OMC frame by solving for the rotation matrix using angular velocity measurements from the gyroscope and the calculated angular velocity from OMC-derived orientation measurements using the singular value decomposition to Wahba’s problem [26].

### 2.3. Error Calculation

The sample-to-sample error is defined as the orientation derived by the fused solution $\left(q_{t}^{GI}\right)$ relative to the orientation derived from the OMC $\left(q_{t}^{GO}\right)$ [27]:

(30) $$q_{t}^{OI}={\left(q_{t}^{GO}\right)}^{C}☉q_{t}^{GI}.$$

Given $q_{t}^{OI}$, error $\left(Τ\right)$ and the error around the Z-axis $\left(\psi \right)$, Y-axis $\left(\theta \right)$, and X-axis $\left(\phi \right)$ at time t are calculated as follows:

(31) $${Τ}_{t}=2\mathrm{acos}\left(q_{0,t}\right)$$

(32) $$\psi_{t}=\mathrm{atan}\left(\frac{2\left(q_{0}q_{3}+q_{1}q_{2}\right)}{q_{0}^{2}+q_{1}^{2}-q_{2}^{2}-q_{3}^{2}}\right)$$

(33) $$\theta_{t}=\mathrm{asin}\left(2\left(q_{0}q_{2}-q_{1}q_{3}\right)\right)$$

(34) $$\phi_{t}=\mathrm{atan}\left(\frac{2\left(q_{0}q_{1}+q_{2}q_{3}\right)}{q_{0}^{2}-q_{1}^{2}-q_{2}^{2}+q_{3}^{2}}\right).$$

Root-mean-square error (RMSE) was calculated to determine average error over a given time period. Given angle α, RMSE can be calculated as follows:

(35) $$RMSE=\sqrt{\frac{\sum \alpha_{i}^{2}}{n}}.$$

### 2.4. Experimental Protocol

Twelve participants (eight females; four males; mean age 25 ± 5 years) were recruited from the University of Alabama, specifically from the Huntsville campus community. Participants were recruited using a convenience sample. They were screened for any self-reported cases of (i) a history of upper body injuries, (ii) any current upper body injuries prior to enrollment, and (iii) the ability to continuously arm cycle for fifteen minutes. Informed consent was provided prior to participation. The University of Alabama in Huntsville Institutional Review Board approved all study procedures.

**Figure 1:**
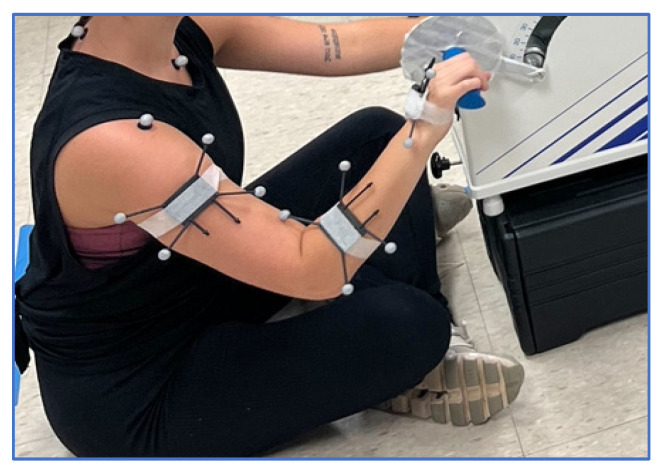
Cycling ergometer trial setup and marker placement.

Participants were asked to perform a continuous arm cycling motion for fifteen minutes on an Ergometer (Monark 881E, Sweden), as seen in Figure 1. Participants were provided visual feedback, including cycle time duration and average revolution per minute (RPM), to maintain self-paced cadence (within 5 RPM of their average) throughout the trial. An IMU (Movella Dot, Netherlands) was placed on the right hand, forearm, and upper arm. A cluster of four reflective markers was rigidly attached to the top of each IMU using double-sided tape for the 10-camera OMC (4× Valkyrie16, 6× Vero2.2 cameras, Vicon, UK) to calculate spatial orientation. The combined OMC/IMC cluster was then taped to the participant in each of the three locations: hand, forearm, and upper arm (Figure 1).

Calibration of the IMCs and OMCs was performed using manufacturer-specified procedures. The OMC global frame was set as the origin in the center of the laboratory space during calibration. Prior to data collection, all three IMUs were time-synchronized following steps specified in the user manual. Both the OMCs and IMUs recorded data at 60 Hz. A static trial and short range of motion (ROM) trial were conducted prior to data collection to verify whether all markers were properly tracked and that the model was working. Once these steps were complete, data was collected simultaneously from the OMCs and IMCs.

### 2.5. Data Processing

All post-processing was accomplished using MATLAB (2024a, MathWorks, Natick, MA, USA). OMC orientation was calculated using the TRIAD method [17]. The angular velocity measurements from the IMU were linearly interpolated to a sampling period of 1/60 s to ensure time synchronization with the OMC. The first two minutes of the cycling trial were used to determine the rotational alignment from the IMU local coordinate frame to the corresponding OMC coordinate frame. Data were subsequently discarded from the dataset (i.e., the same section of the data used to rotationally align the IMU was not used to validate the accuracy of the proposed sensor fusion algorithm). The proposed sensor fusion algorithm was executed once all pairs (i.e., each IMU and corresponding OMC marker cluster) were successfully time-synced and rotationally aligned. For each pair, the algorithm was executed with increasingly longer time intervals from one minute to five minutes in one-minute increments. Initial orientation was set as an identity quaternion $(\left[{1\;0\;0\;0]}^{T}\right)$ and was set to run for ten iterations.

### 2.6. Statistical Analysis

Statistical analyses were performed using the Statistical Package for Social Sciences (SPSS) version 29 (SPSS Statistics for Windows, Version 29.0.2.0 Armonk, NY, USA: IBM Corp.). Intraclass correlation coefficients (ICCs) and 95% confidence intervals (CIs) are presented for the OMC Z-, Y-, and X-axes and the sensor fusion Z-, Y-, and X-axes for the 5 min data collection interval (all sensor placements). A two-way repeated measures analysis of variance (ANOVA) was used to compare the differences in total error over time (1, 2, and 5 min) across the sensor positions to determine if there were differences over time. Statistical significance was set at *p* ≤ 0.05. Data are reported as mean ± standard deviation, mean difference, and 95% CIs.

## 3. Results

### 3.1. Reliability

The ICC and 95% CI values are presented for the OMC Z-, Y-, and X-axes and the sensor fusion Z-, Y-, and X-axes for the 5 min interval of data collection (all sensor placements). The ICC data for the 5 min duration demonstrates the reliability of the algorithm over the longest gap period. All ICCs are reported as having excellent (>0.90) reliability [28] (Table 1).

### 3.2. Total Error

**Figure 2:**
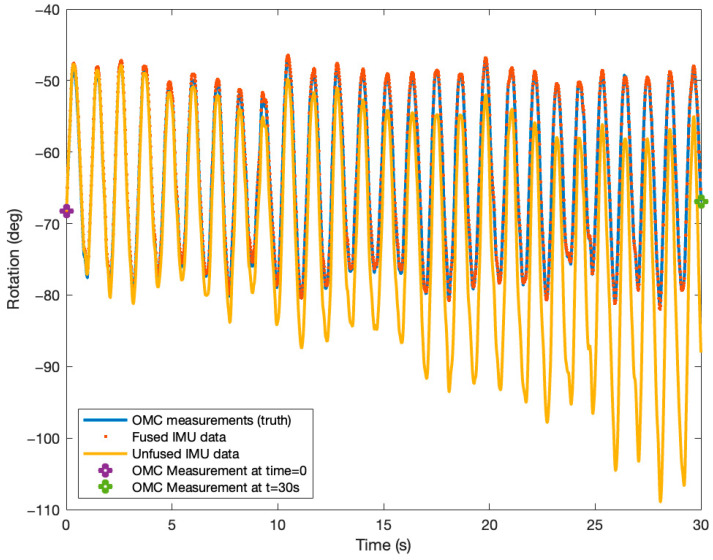
Graph showing the ability of the proposed sensor fusion algorithm to correct for IMU-derived orientation measurements using OMC data at time t = 0 and t = 30 s.

The ability of our sensor fusion algorithm to correct for gyroscope-derived orientation measurements is shown in Figure 2.

The tables below show the average errors for each axis and the average total error across all axes and participants for the 1, 2, and 5 min intervals (values derived from the use of the sensor fusion algorithm). For all sensor placements, average total errors <1.8° were observed across a 5 min duration (Table 2, Table 3 and Table 4).

For the hand sensor (Table 2), the error values for all three axes remained consistent for the 1, 2, and 5 min intervals. An average total error < 1.8° was observed across the 5 min interval. 

For the forearm sensor (Table 3), the error values for all three axes remained consistent for the 1, 2, and 5 min intervals. An average total error < 1.3° was observed across the 5 min interval.

For the upper arm cluster (Table 4), the error values visually appear consistent across the 1, 2, and 5 min intervals. An average total error <1.1° was observed across the 5 min interval.

The studentized residual values showed that no outliers needed to be removed before performing the two-way repeated measures ANOVA (N = 12 for two-way interaction analysis). The Shapiro–Wilk’s test of normality was used to assess whether the two within-subject factors were normally distributed. Some data violated normality and were either positively or negatively skewed, depending on the measurement. Due to the variability of the skewness, the original data were used for the two-way ANOVA analysis. Mauchly’s test of sphericity (*p* > 0.05) indicated that the assumption of sphericity was not violated for the two-way interaction (X^2^ = 16.128, *p* = 0.068). The two-way interaction was not statistically significant between sensor placement and time—F(4, 44) = 1.760, *p* = 0.154, η2 = 0.138.

A main effect of time F(2, 22) = 10.074, *p* < 0.001, η2 = 0.478 was observed. Post hoc analysis with Sidak adjustment for multiple comparisons indicates that min 1 was different from min 5 (mean difference of −0.364; 95% CI −0.594 to −0.134; *p* = 0.003). Statistically significant differences were not observed between min 1 and min 2 (*p* = 0.105) or between min 2 and min 5 (*p* = 0.148). A main effect of sensor placement F(2, 22) = 4.369, *p* = 0.025, η2 = 0.284 was also observed. Post hoc analysis with Sidak adjustment for multiple comparisons indicates that there was no statistical difference between the hand and forearm (*p* = 0.295), the forearm and upper arm (*p* = 0.593), or the hand and upper arm placement (*p* = 0.069).

## 4. Discussion

### 4.1. Reliability of the Algorithm

The ICC can be considered an appropriate test to carry out test comparisons [29]. For this study, the average axis orientation for the OMC, the ‘the gold standard’, was compared to the average axis orientation for the fusion output. An average orientation at a given time interval and placement was determined for both the OMC and fused outputs. With our study design emphasizing longer durations, the ICC results in Table 1 present axis data from all sensor placements for the 5 min interval. These ICCs demonstrate excellent (>0.90) reliability.

### 4.2. OMC-IMC Errors

For all sensor locations, total error < 1.8° was observed over a gap of 5 min without OMC measurements (Table 2, Table 3 and Table 4). Consistent with our hypothesis and the work of Lebel et al. (2013) [19], for a given sensor location, the effects of time on total error were observed (*p* < 0.05); specifically, min 1 was different from min 5. Unexpectedly, an effect of sensor placement on total error was observed, comparing the hand to upper arm placement.

Few studies have performed custom sensor fusion similar to this. One of these studies, conducted by Yang et al. (2021), had reported errors of 4.8° over a 20-second span with sole gyroscope data, which was then further reduced to 2.5° by applying an error-state Kalman filter (ESKF) to the gyroscope data prior to fusing [21]. In our study, average total errors of <1.8° were observed across a 5 min duration (Table 2, Table 3 and Table 4). One specific limitation Yang et al. (2021) was not able to solve for was longer line-of-sight occlusions, which is what this study aimed to address by incorporating trials lasting up to five consecutive minutes [21]. Enayati et al. (2015) used a quaternion-based unscented Kalman filter (UKF) to combine OMC and IMC, and their reported errors were at a maximum of 0.88° for a gap of approximately 10 s due to the OMC recording at 20 Hz [22]. For this study, average total errors were <1.5° across the 2 min interval and <1.8° across the 5 min interval (Table 2). These results demonstrate the feasibility of filling larger line-of-sight marker occlusions and gaps in the data.

There were errors observed that slightly vary based on sensor placement. This has also been observed in several studies assessing the accuracy of IMC-based systems [30,31,32]. Chen et al. (2023), for example, reported errors of 7.3° for the elbow joint and 2.4° for the wrist joint during ‘slow’ movement speed due, in part, to differences in dynamics affecting the accelerometer measurements in IMCs [30]. In our study, we hypothesized that this could be attributed to (i) slightly less accurate local frame alignment between the OMC and IMC for certain sensor placements or (ii) the gyroscope in the IMU being marginally less accurate for certain IMUs.

The results from this study showed that for ≤5 min, all sensor placement measurements were comparable to studies that validated IMCs without the incorporation of OMC measurements. Brodie et al. (2008) used a pendulum swing motion to test the accuracy of IMUs with their own custom fusion algorithm, presenting RMSE between 0.8° and 1.3° [13]. They additionally combined the raw gyroscope data with a Kalman filter given by the IMU manufacturers, which presented RMSE between 8.5° and 11.7°, dependent upon the motion and length of the trial [13]. Robert-Lachaine et al. (2017) reported errors < 7.3° for a given axis [32]. Chen et al. (2023) reported total errors < 10.2°, yielding accuracy improvements due to the sensor fusion algorithm and movement speeds [33]. While these studies have indicated that the proposed fusion approach may not be needed, the errors associated with the IMC-only approach can produce errors significantly higher than those reported here, given that the presence of magnetic disturbance can cause errors up to 180° [34].

### 4.3. Limitations

The main limitation of this study was the motion itself. This specific motion pattern was chosen to provide a repeatable motion that can be recorded for a relatively long period of time in a constrained recording area that minimizes marker occlusion. The motion was easily conducted by participants for the fifteen-minute data collection period. While this worked well for quality OMC data collection, this made it difficult to fully study dynamic movement on all three axes. Therefore, the motion pattern used in this study may only be generalizable to upper-limb cycling motion. Future work should use more dynamic, complex movements for all three axes to demonstrate a more thorough application of the algorithm. More specifically, a manual material handling task could be introduced and studied to mimic a manufacturing process and allow for dynamic movements and processing of joint angles.

Additionally, with this being the foundational work for this kind of research, another limitation was the fact that our research was still being carried out in a laboratory setting. As stated previously, the focus was the algorithm and the foundational fusion work being presented. This facilitated the use of a testing environment that was already equipped with the proper OMC setup to obtain data without any gaps for a comparison with the fused data. Moving forward, one focus of future research will be collecting data in an environment that is outside of the laboratory to combat this limitation and show the true feasibility of this approach. Future iterations can assess how the total number of cameras impacts collection while informing future capture validation for non-laboratory settings.

### 4.4. Implications

This study aimed to demonstrate a custom sensor fusion algorithm that relied on only the first and last frames of OMC data, which were then fused with gyroscope data to fill in the rest. Across the 5 min interval, total errors were between 1.1° and 1.8° depending upon where the sensor was placed on the body (Table 2, Table 3 and Table 4). A slight increase in total error was observed during the longest interval (5 min) and was a jump from the 1 to 5 min interval, thus showing larger gaps in the data.

The results of this study showed the feasibility of using IMU measurements for gap-filling OMC measurements with gap durations upwards of 5 min for cyclic motion patterns. Many similar studies cite their main limitations as the duration of the trial, the lack of human motion, and the test setting environment itself [14,21,22,26]. This study directly alleviated two of those limitations through featuring longer trial durations (i.e., up to 5 min) and the use of human participants. The custom optimization-based sensor fusion method modified from Kok et al. (2017) [24] appeared effective for mitigating OMC marker occlusion.

## 5. Conclusions

This study aimed to create and evaluate an optimization-based sensor fusion algorithm that combines OMC and IMC measurements to provide a more efficient and reliable gap-filling process for OMC measurements to be used for future research. The proposed algorithm takes the first and last frame of OMC data and fills the rest with IMU gyroscope data. In addition to alleviating the time-intensive process of gap-filling, this approach may enable fewer OMC cameras to be used in the future, making it potentially practical for certain field-based motion analysis applications. While it may be impractical to set up a full suite of OMC cameras in a workplace setting, the results of this study suggest that a limited set of OMC cameras (i.e., 3 or 4) could be practical in some applications and that prolonged marker occlusions resulting from using a limited set of cameras can be mitigated by incorporating IMU measurements.

This work can be built upon further by using IMUs to gap-fill OMC data in real time when markers get blocked or other issues occur during data collection in laboratory-based settings, which will directly combat arguably the biggest limitation of using OMCs alone. Additionally, building upon the algorithm will allow for more complex and dynamic movements to be studied and tested in various environments. Future research should also address the limitations of this study. In particular, using more complex movements should be considered to improve the generalizability of the proposed approach. A motion focusing on dynamic motion for all three axes will demonstrate the true feasibility of the algorithm for dynamic movements in other test settings. Ultimately, translating this research from the laboratory to a desired setting is the overarching goal, with the next milestone being conducting this research elsewhere while still producing quality results.

## Figures and Tables

**Table 1:** Intraclass correlation coefficient (ICC) estimates calculated using SPSS based on a mean rating (k = 2), consistency, and a 2-way mixed-effects model.

Placement	Time Interval	ICC	Confidence Interval
Hand Z	5	0.998	(0.993, 0.999)
Hand Y	5	1.000	(0.999, 1.000)
Hand X	5	0.996	(0.987, 0.999)
Forearm Z	5	0.998	(0.994, 0.999)
Forearm Y	5	0.999	(0.998, 1.000)
Forearm X	5	0.995	(0.983, 0.999)
Upper Arm Z	5	0.999	(0.997, 1.000)
Upper Arm Y	5	0.996	(0.987, 0.999)
Upper Arm X	5	0.997	(0.990, 0.999)

**Table 2:** Mean (SD) root-mean-square differences between the fused OMC-IMC data for the hand in degrees (°) compared to OMC data over 1, 2, and 5 min intervals.

Error	1-min	2-min	5-min
X-Axis	1.1 (0.7)	1.4 (0.9)	1.7 (1.0)
Y-Axis	0.3 (0.1)	0.3 (0.2)	0.4 (0.2)
Z-Axis	0.3 (0.2)	0.4 (0.2)	0.6 (0.4)
Total	1.2 (0.7)	1.5 (0.9)	1.8 (1.0)

**Table 3:** Mean (SD) root-mean-square differences between the fused OMC-IMC data for the forearm in degrees (°) compared to OMC data over 1, 2, and 5 min intervals.

Error	1-min	2-min	5-min
X-Axis	0.8 (0.2)	0.8 (0.3)	0.8 (0.3)
Y-Axis	0.4 (0.3)	0.5 (0.3)	0.7 (0.5)
Z-Axis	0.4 (0.2)	0.4 (0.2)	0.6 (0.3)
Total	1.0 (0.3)	1.0 (0.3)	1.3 (0.4)

**Table 4:** Mean (SD) root-mean-square differences between the fused OMC-IMC data for the upper arm in degrees (°) compared to OMC data over 1, 2, and 5 min intervals.

Error	1-min	2-min	5-min
X-Axis	0.5 (0.2)	0.9 (0.3)	0.7 (0.4)
Y-Axis	0.4 (02)	0.5 (0.2)	0.4 (0.2)
Z-Axis	0.6 (0.3)	0.6 (0.3)	0.7 (0.4)
Total	0.9 (0.3)	1.0 (0.3)	1.1 (0.5)

## Data Availability

Data will not be made publicly available since public release was not specified through the Institutional Review Board Approval Process.

## Abbreviations

The following abbreviations are used in this manuscript:

OMC	Optical Motion Capture
IMC	Inertial Motion Capture
IMU	Inertial Measurement Unit
ROM	Range of Motion
SPSS	Statistical Package for Social Sciences
CI	Confidence Interval
ANOVA	Analysis of Variance
ICC	Intraclass Correlation Coefficients
SD	Standard Deviation
ESKF	Error-state Kalman Filter
UKF	Unscented Kalman Filter
RSME	Root-Mean-Square Error
